# USP44 positively regulates innate immune response to DNA viruses through deubiquitinating MITA

**DOI:** 10.1371/journal.ppat.1008178

**Published:** 2020-01-22

**Authors:** Hong-Yan Zhang, Bo-Wei Liao, Zhi-Sheng Xu, Yong Ran, Dong-Peng Wang, Yan Yang, Wei-Wei Luo, Yan-Yi Wang

**Affiliations:** 1 Key Laboratory of Special Pathogens and Biosafety, Wuhan Institute of Virology, Center for Biosafety Mega-Science, Chinese Academy of Sciences, Wuhan, China; 2 University of Chinese Academy of Sciences, Beijing, China; Tufts University, UNITED STATES

## Abstract

Mediator of IRF3 activation (MITA, also known as stimulator of interferon genes, STING) senses the second messenger cyclic GMP-AMP (cGAMP) which is synthesized upon DNA virus infection and activates innate antiviral immune response. It has been demonstrated that the activity of MITA is delicately regulated by various post-translational modifications including polyubiquitination. In this study, we identified the deubiquitinating enzyme USP44 as a positive regulator of MITA. USP44 is recruited to MITA following DNA virus infection and removes K48-linked polyubiquitin moieties from MITA at K236, therefore prevents MITA from proteasome mediated degradation. USP44-deficiency results in acceleration of HSV-1-induced degradation of MITA and reduced induction of type I interferons (IFNs) and proinflammatory cytokines. Consistently, *Usp44*^*-/-*^ mice are more susceptible to HSV-1 infection as indicated by higher tissue viral titers, greater tissue damage and lower survival rate. These findings suggest that USP44 plays a specific and critical role in the regulation of innate immune response against DNA viruses.

## Author summary

Cyclic GMP-AMP synthase (cGAS) senses cytosolic dsDNA and initiates signal transductions, leading to activation of innate immune response. MITA is the key adaptor protein downstream of cGAS and plays a critical role in cGAS-mediated signaling. The activity of MITA is tightly regulated by various post-translational modifications including polyubiquitination and deubiquitination. Here we found that the deubiquitinating enzymes USP44 associates with MITA and removes the K48-linked polyubiquitin chains from MITA, therefore maintains the stability of MITA after DNA virus infection. Deficiency of USP44 results in accelerated degradation of MITA, impaired induction of type I IFNs and proinflammatory cytokines, and increased viral replication. These findings suggest that USP44 is a positive regulator of MITA and plays an important role in the regulation of innate immune response against DNA viruses.

## Introduction

The innate immune response is the first line of host defense against pathogens. Germline-encoded pattern recognition receptors (PRRs) recognize conserved molecular motifs of pathogens called pathogen-associated molecular patterns (PAMPs) and trigger a series of signaling events, leading to induction of type I IFNs, proinflammatory cytokines and downstream antiviral effector proteins, which eventually inhibit the replication of pathogens and eliminate the infected cells [1–4].

Viral nucleic acids act as typical PAMPs that trigger innate immune response. Viral RNAs are recognized by endosomal Toll-like receptors (TLRs) and cytosolic RIG-I-like receptors (RLRs) such as retinoic acid-inducible gene-1 (RIG-I) and melanoma differentiation-associated gene 5 (MDA5) [5–7]. Meanwhile, several proteins have been identified as viral DNA sensors, including Toll-like receptor 9 (TLR9), DNA-dependent activator of IFN-regulatory factors (DAI), RNA polymerase III (Pol-III), IFN-γ-inducible protein 16 (IFI16), DEAD-box helicase 41 (DDX41) and LSM14A [8–13]. However, evidence suggest that these proteins are not universally needed for recognizing viral DNA in various cell types or *in vivo* [14]. In recent years, the nucleotidyltransferase family protein cyclic GMP-AMP (cGAMP) synthase (cGAS) is identified as a cytosolic DNA sensor that induces interferons irrespective of cell type or DNA sequence [15–17]. Upon sensing viral dsDNA, cGAS catalyzes synthesis of cGAMP [16]. cGAMP then binds to and activates adaptor protein MITA (also known as endoplasmic reticulum (ERIS), MPYS and STING), which is located on the endoplasmic reticulum (ER) membrane [18–23]. Once associated with cGAMP, MITA traffics from ER through Golgi apparatus to perinuclear microsomal compartments [19, 24, 25]. During this process, MITA recruits the TANK-binding kinase 1 (TBK1) and is phosphorylated by TBK1, which is important for MITA to recruit interferon regulatory factor 3 (IRF3) [18, 24]. IRF3 undergoes phosphorylation by TBK1. Phosphorylated IRF3 form dimers and translocate to the nucleus, leading to the induction of type I IFNs and downstream effector genes [26, 27].

As a key adaptor protein in innate immune response against DNA viruses, the activity of MITA is delicately regulated. Several post-translational modifications, such as phosphorylation, sumoylation and polyubiquitination, have been reported to play important roles in regulation of MITA [28]. For example, phosphorylation of MITA at Ser358 and Ser366 is crucial for its activation and recruitment of IRF3 [18, 26, 27]; sumoylation of MITA in the early phase of viral infection by TRIM38 promotes its stability and activation whereas desumoylation of MITA in the late phase by SENP2 leads to its degradation thus avoiding sustained activation of MITA [29]. In addition, polyubiquitination of MITA have also been reported to distinctly regulate its activity. The E3 ubiquitin ligases TRIM56 and TRIM32 mediated K63-linked polyubiquitination of MITA and AMFR mediated K27-linked polyubiquitination of MITA have been shown to enhance the antiviral innate immune response [30–32]. RNF5 catalyzes K48-linked polyubiquitination of MITA that results in its degradation [33], and this could be inhibited by RNF26 mediated K11-linked polyubiquitination of MITA at the same site [34].

Deubiquitination is a reverse biochemical process of polyubiquitination, in which polyubiquitin chains previously added to target proteins are removed by deubiquitinating enzymes (DUBs). Recently, several DUBs have been reported to regulate the antiviral innate immune response. For example, USP14 removes K48-linked polyubiquitination of cGAS therefore impairs autophagic degradation of cGAS [35]. It has been reported that USP13 removes K27-linked polyubiquitin chains from MITA and inhibits interaction between MITA and TBK1, resulting in impaired antiviral innate immune response [36]. USP49 has been shown to deconjugate K63-linked polyubiquitin chains from MITA and inhibits the aggregation and activation of MITA [37].

In this study, we screened for DUBs that involve in DNA virus-triggered induction of type I IFNs and identified ubiquitin-specific protease 44 (USP44) as a positive regulator. USP44 consists of 712 amino acids, which contains a Zn-finger domain at the N-terminus and a USP domain at the C-terminus [38]. USP44 has been shown to be involved in many cellular processes, including embryonic stem cell (ESC) differentiation, cell proliferation, DNA repair and tumor progression [39–43]. However, it is dispensable for the differentiation of hematopoietic stem cells (HSC) and barely affects the development of immune cells [44]. So far, whether USP44 is involved in immune response is unclear. We found that overexpression of USP44 potentiated DNA- but not RNA virus-triggered production of type I IFNs and proinflammatory cytokines. Conversely, USP44 deficiency suppressed cytosolic DNA- and DNA virus-triggered innate immune response. Further study revealed that USP44 was recruited to MITA and selectively removed K48-linked polyubiquitin chains from MITA at K236, therefore inhibited proteasome-mediated degradation of MITA and promoted antiviral response against DNA viruses. Our study shed new light on the function of USP44 and further demonstrated the importance of deubiquitination in the regulation of antiviral innate immune response.

## Results

### USP44 positively regulates DNA virus-induced innate immune response

**Fig 1:**
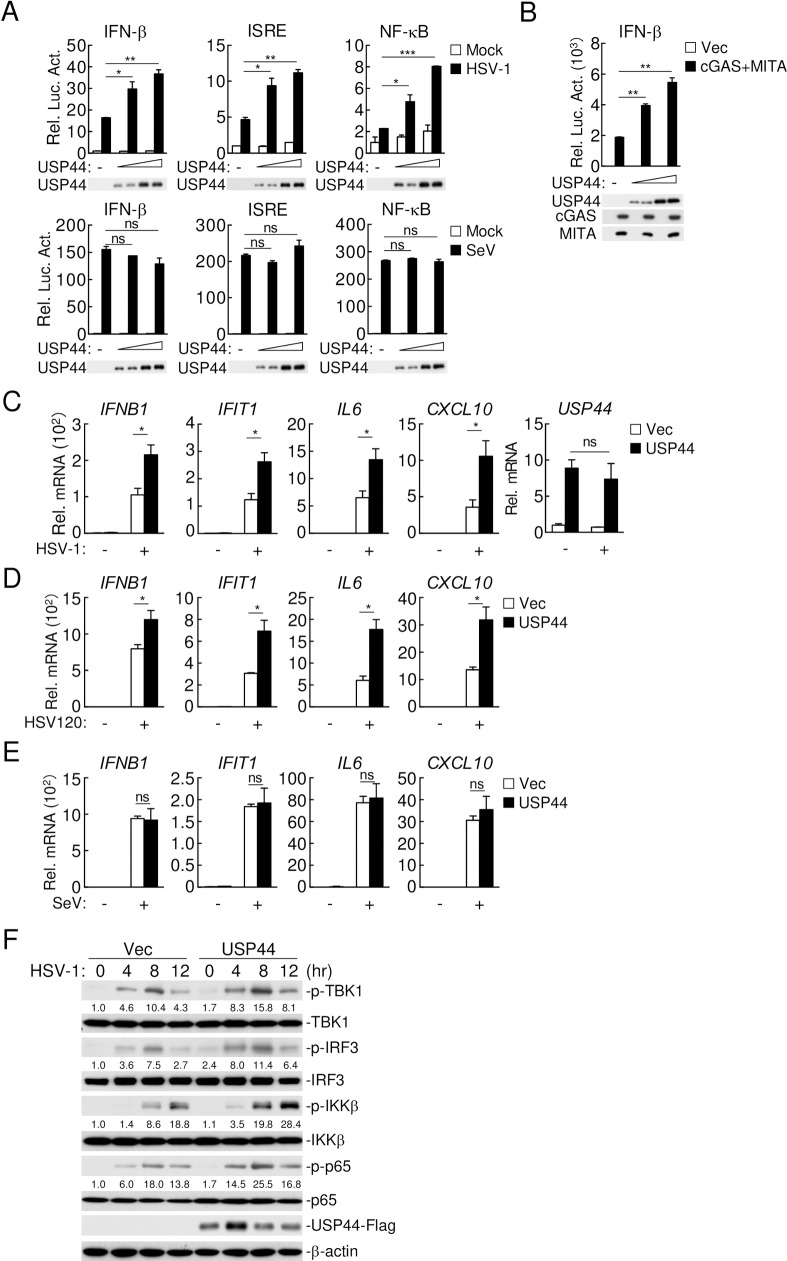
USP44 positively regulates DNA virus-triggered signaling. **(A)** HEK293 cells (1 x 10^5^) were transfected with IFN-β (0.05 μg), ISRE (0.05 μg) or NF-κB (0.01 μg) reporter plasmids plus empty vector or increased amounts of USP44 expression plasmids. Twenty-four hours after transfection, cells were left uninfected, infected with HSV-1 (top) or SeV (bottom) (MOI = 1) for 12 hours before luciferase assays were performed. **(B)** HEK293T cells (1 x 10^5^) were co-transfected with empty vector or cGAS and MITA, IFN-β reporter (0.05 μg), and increased amounts of USP44 for 24 hours before luciferase assays were performed. **(C and D)** THP-1 cells (4 x 10^5^) stably expressing USP44 were left untreated, infected with HSV-1 (MOI = 1) (C) or transfected with HSV120 (2 μg/ml) (D) for 12 hours before qPCR analysis. **(E)** THP-1 cells (4 x 10^5^) stably expressing USP44 were left uninfected or infected with SeV (MOI = 1) for 12 hours before qPCR analysis. **(F)** THP-1 cells (4 x 10^5^) stably expressing USP44 were left uninfected or infected with HSV-1 (MOI = 1) for the indicated times followed by immunoblot analysis. Graphs show mean ± S.D. n = 3. *P < 0.05, **P < 0.01 (Student’s *t*-test).

To identify potential molecules involved in DNA-triggered signaling. We screened ~40 independent human DUB cDNA expression plasmids for their abilities to regulate the *IFNB* promoter in reporter assays. These efforts led to identification of USP44 as a candidate protein that could potentiate activation of the *IFNB* promoter triggered by the DNA virus herpes simplex virus 1 (HSV-1) (S1 Fig). Overexpression of USP44 potentiated HSV-1-, but not SeV-induced activation of the *IFNB* promoter, interferon-stimulated response element (ISRE) and NF-κB in a dose-dependent manner in HEK293 cells (Fig 1A). Consistently, overexpression of USP44 enhanced cGAS-MITA-induced activation of the *IFNB* promoter in a dose-dependent manner (Fig 1B). We next established a THP-1 cell line stably expressing USP44. qPCR experiments indicated that USP44 enhanced transcription of *IFNB1*, *IFIT1*, *IL6* and *CXCL10* genes in response to HSV-1 (Fig 1C). In addition, transcription of downstream genes induced by transfected HSV120 (120-mer dsDNA representing the genome of HSV-1) was also enhanced in USP44-expressing THP-1 cells (Fig 1D). In contrast, the transcription of downstream genes induced by SeV was not markedly affected in USP44-expressing THP-1 cells (Fig 1E). Moreover, USP44 enhanced HSV-1-induced phosphorylation of TBK1, IRF3, IKKβ and p65 (Fig 1F). These results suggest that USP44 specifically regulates in DNA virus-triggered innate immune response.

### Knockdown of USP44 inhibits DNA virus-triggered signaling

**Fig 2:**
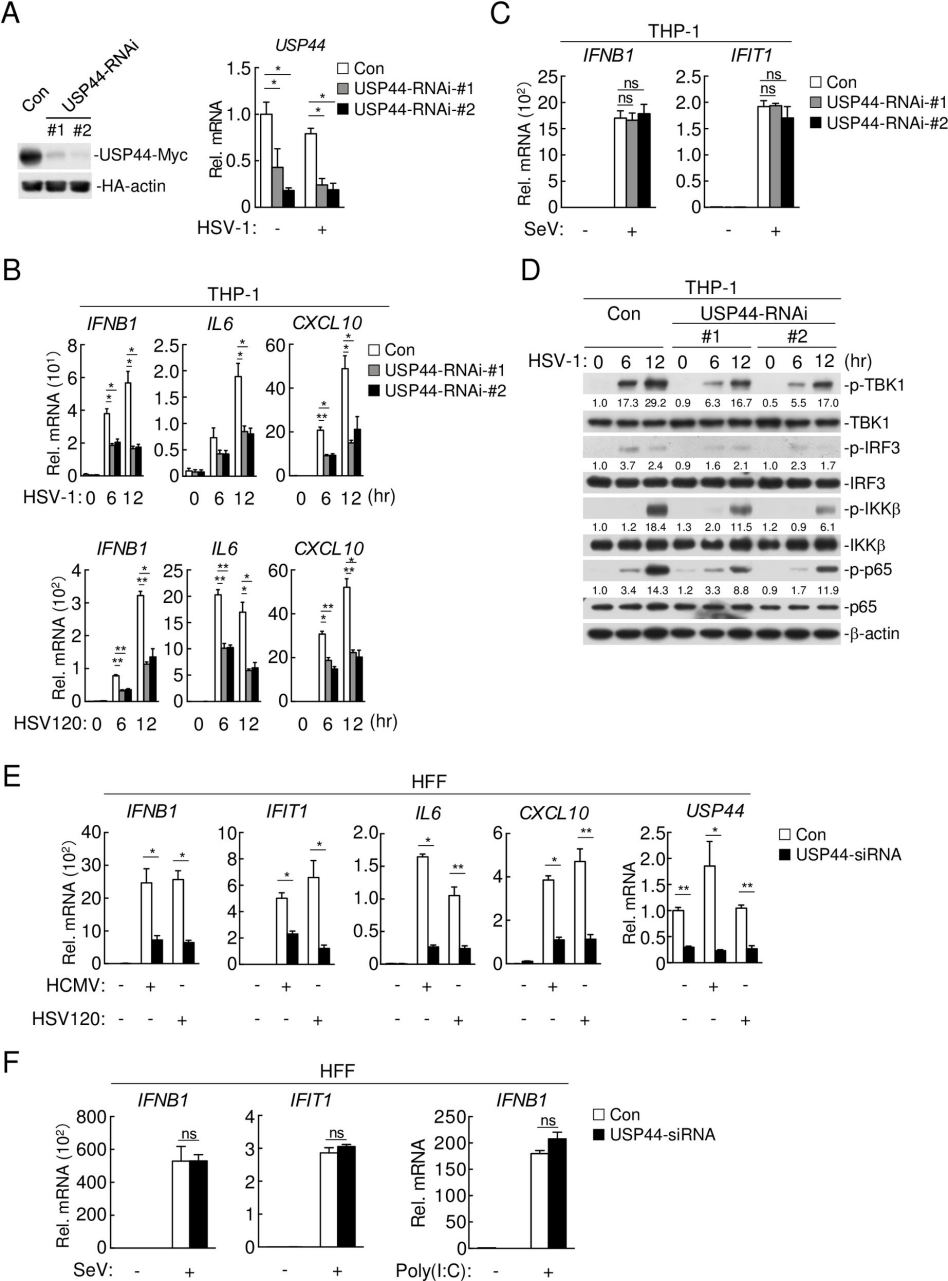
Knockdown of USP44 inhibits DNA virus-induced innate immune responses. **(A)** (Left) HEK293T cells (4 x 10^5^) were co-transfected with USP44-Myc, HA-actin (0.1 μg each) and control- or indicated USP44-RNAi plasmids (1μg each). Twenty-four hours after transfection, cell lysates were analyzed by immunoblotting with anti-Myc or anti-HA antibodies. (Right) THP-1 cells (4 x 10^5^) stably expressing USP44-RNAi were infected with HSV-1 (MOI = 1) for 12 hours before qPCR analysis. **(B)** THP-1 cells (4 x 10^5^) stably expressing USP44-RNAi were infected with HSV-1 (MOI = 1) (top) or transfected with HSV120 (2 μg/ml) (bottom) for the indicated times followed by qPCR analysis. **(C)** THP-1 cells (4 x 10^5^) stably expressing USP44-RNAi were left uninfected or infected with SeV (MOI = 1) for 12 hours before qPCR analysis. **(D)** THP-1 cells (4 x 10^5^) stably expressing USP44-RNAi were infected with HSV-1 (MOI = 1) for the indicated times and analyzed by immunoblotting with the indicated antibodies. **(E)** HFF cells (4 x 10^5^) were transfected with the indicated control-siRNA or USP44-siRNA. Thirty-six hours later, cells were left untreated, infected with HCMV (MOI = 1) or transfected with HSV120 (2 μg/ml) for 12 hours before qPCR analysis. **(F)** HFF cells (4 x 10^5^) were transfected with the indicated USP44-siRNA or control-siRNA. Thirty-six hours later, cells were left untreated, infected with SeV (MOI = 1) (left) or transfected with poly(I:C) (2 μg/ml) (right) for 12 hours before qPCR analysis. Graphs show mean ± S.D. n = 3. *P < 0.05, **P < 0.01 (Student’s *t*-test).

To confirm the role of endogenous USP44 in DNA virus-triggered signaling, we designed two pSuper-RNAi constructs which could efficiently knockdown the expression of USP44 (Fig 2A). qPCR experiments indicated that transcription of *IFNB1*, *IL6* and *CXCL10* induced by HSV-1 infection and transfected dsDNA, such as HSV120 and interferon stimulating DNA (ISD), was markedly reduced in USP44-RNAi expressing THP-1 cells compared with control cells (Fig 2B and S2 Fig). In contrast, knockdown of USP44 had little effects on SeV-induced the transcription of downstream genes in THP-1 cells (Fig 2C). Knockdown of USP44 also reduced HSV-1-induced phosphorylation of TBK1, IRF3, IKKβ and p65 in THP-1 cells (Fig 2D).

To determine whether USP44 regulates in a cell- and virus-specific manner, we used independent siRNA construct to knockdown USP44 in human foreskin fibroblasts (HFFs), which are permissive to human cytomegalovirus (HCMV) infection. We found that knockdown of USP44 inhibited HCMV- and HSV120-induced transcription of *IFNB1*, *IFIT1*, *IL6* and *CXCL10* genes (Fig 2E), but had no marked effects on SeV- or dsRNA mimic poly(I:C)-induced transcription of antiviral genes in HFFs (Fig 2F). These results suggest that USP44 plays a general role in modulating innate immune response to DNA virus in various cell types.

### USP44-deficiency impairs innate immune response to DNA viruses in mice

**Fig 3:**
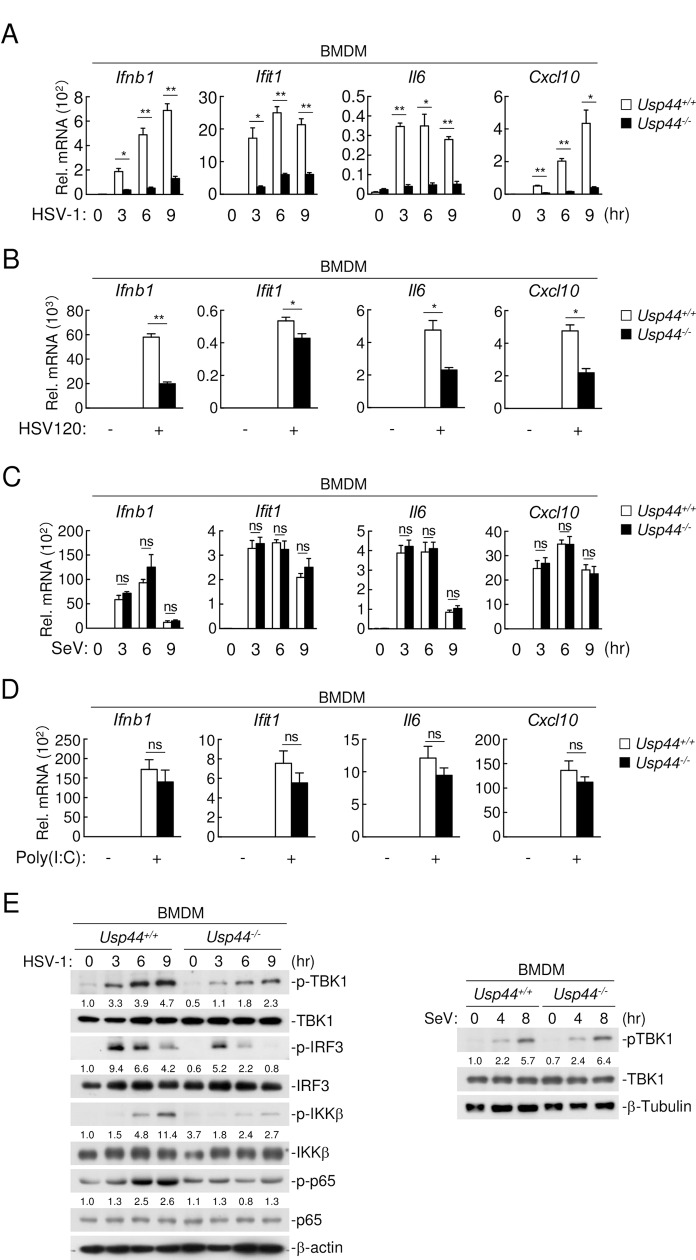
Usp44 is required for DNA virus-triggered induction of antiviral genes. **(A and B)** *Usp44*^*+/+*^ and *Usp44*^*-/-*^ BMDMs (4 x 10^5^) were infected with HSV-1 (MOI = 1) for the indicated times (A) or transfected with HSV120 (2 μg/ml) for 6 hours (B), followed by qPCR analysis. **(C and D)** *Usp44*^*+/+*^ and *Usp44*^*-/-*^ BMDMs (4 x 10^5^) were infected with SeV (MOI = 1) for the indicated times (C) or transfected with poly(I:C) (2 μg/ml) for 6 hours (D), followed by qPCR analysis. **(E)** *Usp44*^*+/+*^ and *Usp44*^*-/-*^ BMDMs (4 x 10^5^) were left uninfected, infected with HSV-1 (MOI = 1) (left) or SeV (MOI = 1) (right) for the indicated times followed by immunoblotting with the indicated antibodies. Graphs show mean ± S.D. n = 3. *P < 0.05, **P < 0.01 (Student’s *t*-test).

To further investigate the roles of USP44 *in vivo*, we used *Usp44* knockout mice (S3A Fig). *Usp44* mRNA was lowly expressed in wild-type bone marrow-derived macrophages (BMDMs) and murine lung fibroblasts (MLFs), but was up-regulated following HSV-1 infection. In these cells, *Usp44* mRNA was not detectable either before or after HSV-1 infection in *Usp44*^*-/-*^ cells (S3B Fig). qPCR experiments indicated that transcription of *Ifnb1*, *Ifit1*, *Il6* and *Cxcl10* genes induced by HSV-1 and Vaccinia virus (VACV) infection was significantly dampened in *Usp44*^*-/-*^ compared with *Usp44*^*+/+*^ BMDMs (Fig 3A and S3C Fig). In addition, transcription of downstream genes induced by transfected HSV120 and ISD was also inhibited in BMDMs (Fig 3B and S3D Fig). In contrast, USP44-deficiency showed no effects on the transcription of antiviral genes induced by SeV or transfected poly(I:C) (Fig 3C and 3D). Consistently, HSV-1-induced phosphorylation of TBK1, IRF3, IKKβ and p65, but not SeV-induced phosphorylation of TBK1, was dramatically impaired in *Usp44*^*-/-*^ BMDMs (Fig 3E). Similar results were also found in *Usp44*^*-/-*^ mouse lung fibroblasts (MLFs) (S3E–S3G Fig).

**Fig 4:**
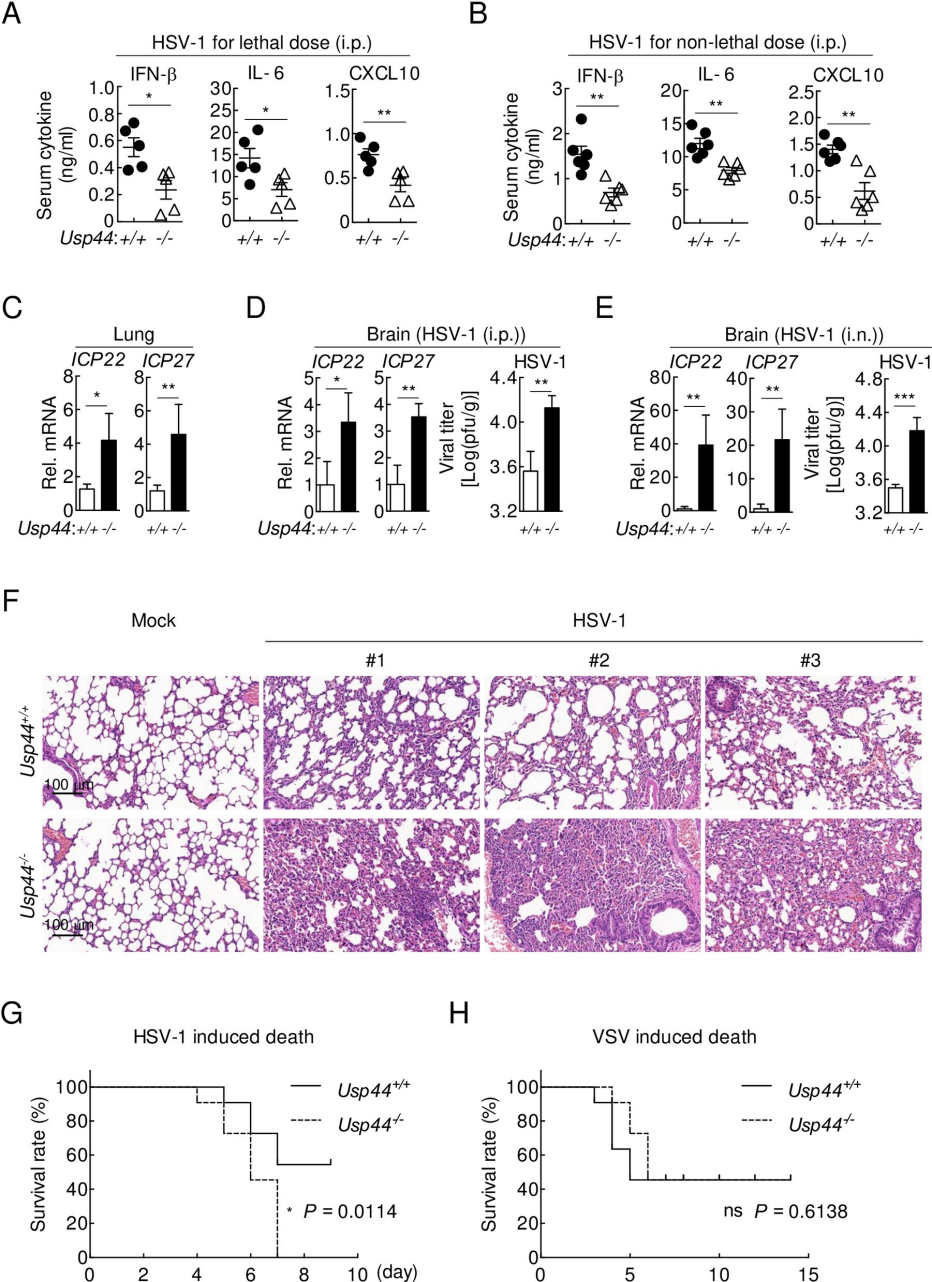
USP44 is critical for host defense against DNA viruses. **(A and B)** *Usp44*^*+/+*^ and *Usp44*^*-/-*^ mice (8 weeks old) were intra-peritoneally (i.p.) injected with HSV-1 (3 x 10^7^ PFU per mouse, n = 5) (A) or HSV-1 (3 x 10^6^ PFU per mouse, n = 6) (B) for 6 hours before ELISA were performed with the sera. Each symbol represents an individual mouse. **(C)** *Usp44*^*+/+*^ and *Usp44*^*-/-*^ mice (8 weeks old, n = 3) were i.p. injected with HSV-1 (3 x 10^7^ PFU per mouse). The lung mRNA levels of the indicated genes were quantified by qPCR at 36 hours post infection. **(D and E)** *Usp44*^*+/+*^ and *Usp44*^*-/-*^ mice (8 weeks old) were i.p. injected with HSV-1 (3 x 10^7^ PFU per mouse, n = 3) (D) or i.n. inoculated with HSV-1 (3 x 10^6^ PFU per mouse, n = 4) (E). Five days later, the brains were collected, mRNA levels of HSV-1 *ICP22* and *ICP27* (left), and HSV-1 viral titers (right) were measured by qPCR analysis and plaque assays respectively. **(F)** *Usp44*^*+/+*^ and *Usp44*^*-/-*^ mice (8 weeks old, n = 3) were i.p. injected with HSV-1 (3 x 10^7^ PFU per mouse) for 5 days and lung sections were analyzed by H&E staining. Scale bars, 100 μm. **(G and H)** *Usp44*^*+/+*^ and *Usp44*^*-/-*^ mice (8 weeks old, n = 11) were i.p. injected with HSV-1 (3 x 10^7^ PFU per mouse) (G) or VSV (1 x 10^8^ PFU per mouse) (H). Survival of these mice were monitored daily for the indicated days. **Data are representative of three independent experiments.** *P < 0.05, **P < 0.01 (Student’s *t* test for A-C and Log-rank test for E).

To investigate whether USP44 is important for host antiviral response *in vivo*, wild-type and *Usp44* knockout mice were intra-peritoneally infected with HSV-1 at lethal or non-lethal dose. While the concentrations of serum cytokines including IFN-β, IL-6 and CXCL10 were obviously lower in *Usp44*^*-/-*^ than that in the wild type mice (Fig 4A and 4B), the lung and brain levels of HSV-1 *ICP22* and *ICP27* mRNA as well as the brain viral titre were significantly increased in *Usp44*^*-/-*^ mice (Fig 4C and 4D). Similar results were obtained when mice are intra-nasally infected (Fig 4E). Furthermore, hematoxylin and eosin staining indicated that the lung tissues of *Usp44*^*-/-*^ mice exhibited greater infiltration of immune cells and tissue damage after HSV-1 infection compared with that of *Usp44*^*+/+*^ mice (Fig 4F). Consistently, *Usp44*^*-/-*^ mice were more susceptible to HSV-1-, but not VSV-induced death compared to their wild-type littermates (Fig 4G and 4H). Collectively, these data suggest that USP44 is essential for host defense against DNA virus infection.

### USP44 acts at the level of MITA

**Fig 5:**
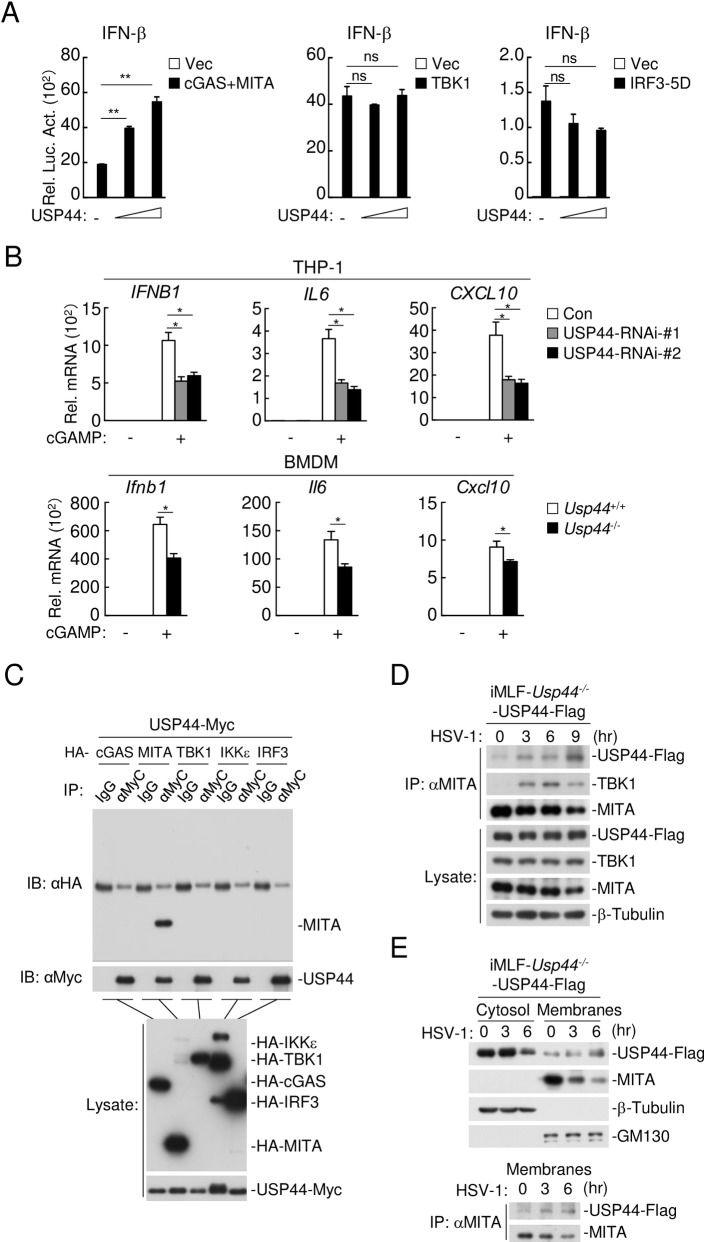
USP44 acts at the level of MITA. **(A)** HEK293T cells (1 x 10^5^) were co-transfected with IFN-β reporter (0.05 μg), the indicated expression plasmids (0.05 μg each) and the increased amounts of USP44 expression plasmids. Twenty-four hours later, cells were analyzed by luciferase assays. **(B)** THP-1 cells (4 x 10^5^) knocked down of USP44 (top) or murine BMDM cells (4 x 10^5^) deficient of USP44 (bottom) were left untreated or treated with cGAMP (0.2 μg/ml) for 4 hours before qPCR analysis. **(C)** HEK293T cells (2 x 10^6^) were transfected with the indicated plasmids (5 μg each) for 24 hours before co-immunoprecipitation and immunoblot were performed. **(D)** *Usp44*^*-/-*^ iMLFs (3 x 10^7^) reconstituted with USP44-Flag were infected with HSV-1 (MOI = 1) for the indicated times in the presence of MG132 (10 μM), followed by immunoprecipitation with anti-MITA and immunoblot with the indicated antibodies. **(E)** *Usp44*^*-/-*^ immortalized MLFs (3 x 10^7^) reconstituted with USP44-Flag were infected with HSV-1 (MOI = 1) for the indicated times. The subcellular fractions were analyzed by immunoblotting with the indicated antibodies (top). The membraneous fraction was analyzed by co-immunoprecipitation and immunoblot with the indicated antibodies (bottom). Graphs show mean ± S.D. n = 3. *P < 0.05, **P < 0.01 (Student’s *t*-test).

To investigate the mechanisms on how USP44 modulates innate immune responses to DNA viruses, we examined the effects of USP44 on activation of the *IFNB* promoter mediated by components of the DNA-triggered signaling pathways. Reporter assays indicated that co-transfection of USP44 enhanced activation of the *IFNB* promoter mediated by cGAS and MITA but had no marked effects on activation of the *IFNB* promoter mediated by their downstream components TBK1 and IRF3-5D (a constitutively active mutant of IRF3) (Fig 5A). Interestingly, knockdown of USP44 in THP-1 cells or knockout of *Usp44* in mouse BMDMs inhibited cGAMP-triggered transcription of downstream genes (Fig 5B). These results indicate that USP44 acts downstream of cGAMP and upstream of TBK1-IRF3. Consistently, transient transfection and coimmunoprecipitation experiments showed that USP44 interacted with MITA but not cGAS, TBK1, IKKε or IRF3 (Fig 5C). Unfortunately, after extensive efforts, our study, as well as the published studies on USP44 [39–42, 44] have not been able to identify a USP44 antibody that can detect endogenous USP44 protein. To overcome this obstacle, we reconstituted immortalized *Usp44*^*-/-*^ MLF cells (iMLFs) with Flag-tagged USP44 (USP44-Flag) by retrovirus-mediated transduction. Using this cell line, we found that USP44 was associated with MITA after HSV-1 infection (Fig 5D). USP44 have been reported to mainly localize in the nucleus [38, 43]. However, in the subcellular fractionation experiments, USP44 was detected in both the nuclear and cytoplasmic fractions (S4 Fig). Since MITA is a transmembrane protein [19, 21], we further examined whether USP44 was recruited to membranes during HSV-1 infection. As shown in Fig 5E (top panel), while USP44 was detected in both the membraneous and cytosolic fractions in uninfected cells, it migrated from cytosol to membranes after HSV-1 infection (Fig 5E, top panel). Importantly, co-immunoprecipitation experiments with the membraneous fraction demonstrated that USP44 associated with MITA on membranes (Fig 5E, bottom panel). Taken together, these results suggest that USP44 is recruited to membrane-localized MITA after HSV-1 infection.

### USP44 deubiquitinates and stabilizes MITA

**Fig 6:**
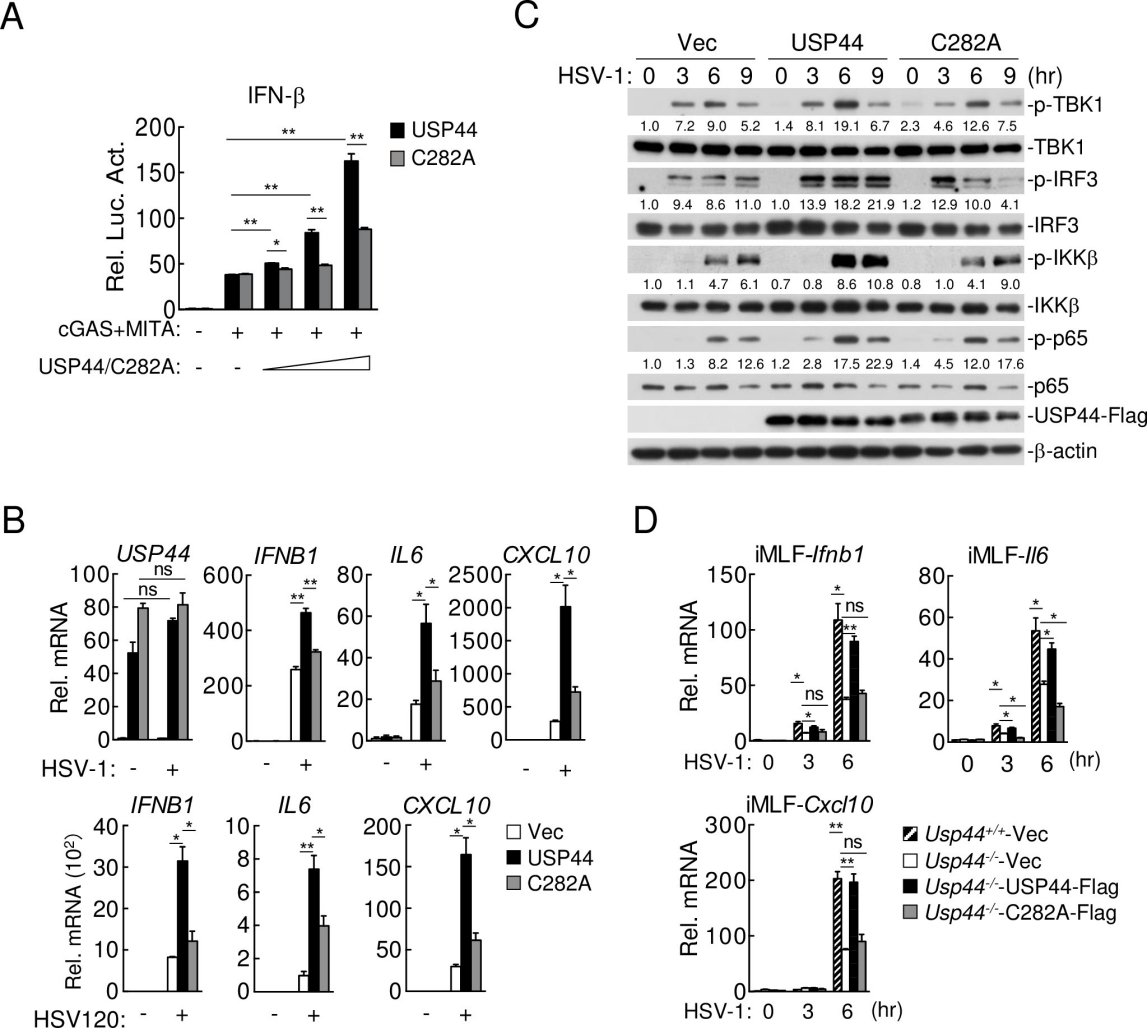
The DUB activity is required for USP44 to regulate antiviral response. **(A)** HEK293T cells (1 x 10^5^) were co-transfected with cGAS (0.01 μg), MITA (0.04 μg), *IFNB* promoter (0.05 μg) and increased amounts of USP44 or USP44(C282A) for 24 hours before luciferase assays were performed. **(B)** THP-1 cells (4 x 10^5^) stably expressing empty vector, USP44 or USP44(C282A) were left uninfected, infected with HSV-1 (MOI = 1) (top) or transfected with HSV120 (2 μg/ml) (bottom) for 12 hours before qPCR analysis. **(C)** THP-1 cells (4 x 10^5^) stably expressing empty vector, USP44 or USP44(C282A) were infected with HSV-1 (MOI = 1) for the indicated times, followed by immunoblot with the indicated antibodies. **(D)** *Usp44*^*+/+*^ and *Usp44*^*-/-*^ iMLFs (4 x 10^5^) reconstituted with empty vector (Vec), USP44-Flag or USP44(C282A)-Flag were left uninfected or infected with HSV-1 (MOI = 1) for the indicated times, followed by qPCR analysis. Graphs show mean ± S.D. n = 3. *P < 0.05, **P < 0.01 (Student’s *t*-test).

Since USP44 is a DUB, we determined whether USP44 functions by deubiquitinating MITA. Reporter assays indicated that USP44(C282A), a deubiquitinase inactive mutant of USP44, lost its ability to enhance cGAS-MITA-mediated activation of the *IFNB* promoter (Fig 6A). qPCR experiments showed that USP44 but not USP44(C282A) promoted transcription of downstream effector genes induced by HSV-1 infection or transfected HSV120 in THP-1 cells (Fig 6B). Consistently, USP44(C282A) also lost its ability to potentiate phosphorylation of TBK1, IRF3, IKKβ and p65 triggered by HSV-1 infection (Fig 6C). To further confirm whether the deubiquitinase activity of USP44 was required for its function, we reconstituted *Usp44*^*-/-*^ iMLFs with wild-type USP44 or USP44(C282A) respectively. qPCR results showed that HSV-1-induced transcription of *Ifnb1*, *Il6* and *Cxcl10* genes was rescued by reconstitution of USP44 but not USP44(C282A) (Fig 6D). These results suggest that the enzymatic activity of USP44 is essential for its regulation of innate immune response to DNA viruses.

**Fig 7:**
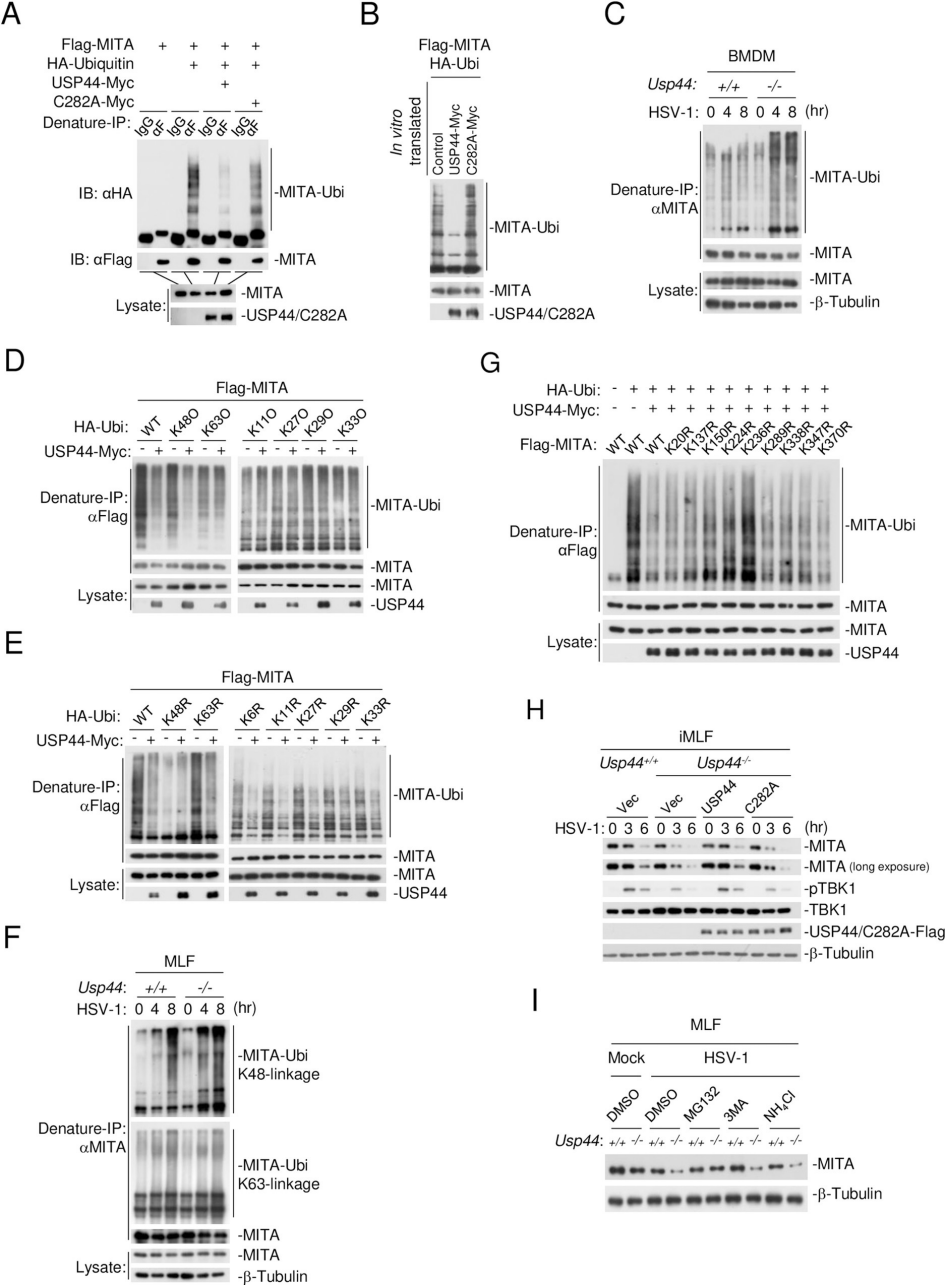
USP44 stabilizes MITA by selectively removing K48-linked polyubiquitin chains from MITA at K236. **(A)** HEK293T cells (2 x 10^6^) transfected with the indicated plasmids for 24 hours before deubiquitination assays were performed. **(B)** *In vitro* deubiquitination assays were performed with ubiquitinated MITA and the indicated *in vitro* generated proteins. The samples were then analyzed by immunoblot with the indicated antibodies. **(C)** *Usp44*^*+/+*^ and *Usp44*^*-/-*^ BMDMs (3 x 10^7^) were left uninfected or infected with HSV-1 (MOI = 1) for the indicated times in the presence of MG132 (10 μM) before deubiquitination assays were performed. **(D and E)** HEK293T cells (1 x 10^6^) were transfected with Flag-MITA and the indicated plasmids for 24 hours before deubiquitination assays were performed. **(F)** *Usp44*^*+/+*^ and *Usp44*^*-/-*^ MLFs (3 x 10^7^) were left uninfected or infected with HSV-1 (MOI = 1) for the indicated times in the presence of MG132 (10 μM) before deubiquitination assays were performed. **(G)** HEK293T cells (1 x 10^6^) were transfected with Flag-MITA or MITA mutants with the indicated plasmids for 24 h before deubiquitination assays were performed. **(H)** *Usp44*^*+/+*^ and *Usp44*^*-/-*^ iMLFs (4 x 10^5^) reconstituted with empty vector (Vec), USP44-Falg or USP44(C282A)-Flag were left uninfected or infected with HSV-1 (MOI = 1) for the indicated times, followed by immunoblot with the indicated antibodies. **(I)** MLFs (4 x 10^5^) were left uninfected or infected with HSV-1 (MOI = 1) in the presence or absence of MG132 (10 μM), 3-MA (500 ng/ml) or NH_4_Cl (25 mM) for 6 hours and then analyzed by immunoblot with the indicated antibodies.

We next determined whether USP44 can deubiquitinate MITA. We found that USP44 but not USP44(C282A) caused deubiquitination of MITA (Fig 7A). *In vitro* experiments confirmed that USP44 but not USP44(C282A) directly deubiquitinated MITA (Fig 7B). Conversely, USP44-deficiency increased HSV-1-induced polyubiquitination of MITA in BMDMs (Fig 7C).

To further examine which type of polyubiquitin moieties USP44 removes from MITA, we performed deubiquitination assays with wild-type ubiquitin and the K-only mutants of ubiquitin including K11O, K27O, K29O, K33O, K48O and K63O, in which only the indicated lysine residue was retaining. As shown in Fig 7D, USP44 selectively removed K48- but not other linkage-mediated polyubiquitin moieties from MITA (Fig 7D). In addition, deubiquitination assays with ubiquitin mutants K6R, K11R, K27R, K29R, K33R, K48R and K63R, in which only the indicated lysine residue was mutated to arginine, showed that USP44 impaired MITA ubiquitination of all tested linkages but failed to deubiquitinate MITA when the K48 residue of ubiquitin was mutated (Fig 7E), indicating that USP44 specifically removes K48-linked polyubiquitin chains from MITA. To further confirm this conclusion, we examined the level of K48- and K63-linked ubiquitination of endogenous MITA induced by HSV-1 infection in WT and *Usp44*^*-/-*^ cells. The results showed that knockout of USP44 potentiated K48- but not K63-linked ubiquitination of endogenous MITA (Fig 7F).

Next we investigated which residue(s) of MITA was targeted by USP44. We performed deubiquitination assays with MITA and a series of its KR mutants where the lysine residues have been individually mutated to arginine. The results showed that among all the KR mutants of MITA, the K236R mutant was resistant to USP44-mediated deubiquitination while the K150R and K224R mutants also exhibited minimal resistance, suggesting that USP44 mainly deubiquitinates MITA at K236 (Fig 7G). Taken together, these findings suggest that USP44 mainly deconjugates K48-linked polyubiquitin chains from MITA at K236.

It has been shown that viral infection-triggered K48-polyubiquitination of MITA promotes its degradation by proteosomes [33, 45–47]. As shown in Fig 7H & 7I, HSV-1 induced degradation of MITA was aggravated in *Usp44*^*-/-*^ MLFs compared with the wild-type cells. Importantly, such aggravation was rescued by reconstitution of *Usp44*^*-/-*^ MLFs with USP44 but not USP44(C282A), or by proteasomal but not autophagic or lysosomal inhibitors (Fig 7H and 7I). Taken together, these findings indicate that USP44 prevents MITA from undergoing proteasome-mediated degradation by removing K48-linked polyubiquitination chains at K236 of MITA after DNA virus infection.

### USP44-mediated regulation of cGAS-MITA signaling is independent of USP20 and CYLD

It has been reported that the deubiquitin enzymes USP20 and CYLD could remove K48-linked polyubiquitin moieties from MITA [48, 49]. Therefore, we further investigated the relevance of USP44, USP20 and CYLD in their regulation of MITA-mediated signaling. As shown in S5A and S5B Fig, USP44 cooperated with USP20 or CYLD to further enhance cGAS-MITA-mediated activation of *IFNB* promoter, ISRE and NF-κB, as well as to further remove K48-linked polyubiquitin chains from MITA (S5A and S5B Fig). In reconstitution experiments, HSV-1-triggered transcription of *Ifnb1*, *Il6* and *Cxcl10* genes was rescued by reconstitution of USP44 but not the enzymatically inactive mutant C282A, USP20, or CYLD in *Usp44*^*-/-*^ iMLFs (S5C Fig). These data indicate that USP44-mediated regulation of MITA is independent of USP20 or CYLD.

## Discussion

The cGAS-MITA pathway plays an important role in innate immune response to DNA viruses [15, 16, 18, 19, 24]. This pathway is tightly regulated so that the host can efficiently initiate innate antiviral response and timely terminate it to avoid immune dysfunctions. Various post-translationally modifications, including different types of polyubiquitination, play critical roles in regulation of cGAS-MITA-mediated innate immune response. In this study, we identified USP44 as a positive regulator of innate immune response to DNA viruses by deubiquitinating and stabilizing MITA after viral infection.

Overexpression of USP44 increased cytosolic DNA- and DNA virus-triggered activation of downstream effector genes whereas knockdown of USP44 inhibited HSV-1-induced transcription of downstream effector genes in various cell types. Consistently, USP44-deficiency impaired HSV-1- and cytosolic DNA-induced transcription of downstream genes in murine immune cells. Furthermore, *Usp44*^*-/-*^ mice showed lower serum cytokines levels, higher viral loads in lungs and brains after HSV-1 infection, and were more susceptible to HSV-1-induced death. In these experiments, USP44 did not affect innate immune response to the RNA viruses SeV, VSV or cytosolic dsRNA, suggesting that USP44 plays a specific role in modulating innate immune response to DNA viruses.

USP44 interacted with MITA following HSV-1 infection. Although previous studies have reported that USP44 is mainly localized in nucleus[38, 43], subcellular fractionation experiments showed that USP44 is distributed in both nucleus and cytoplasm. Interestingly, a portion of cytoplasmic USP44 migrated to membranes after viral infection. Given that MITA is a transmembrane protein and that the MITA-USP44 interaction was detected in the membraneous fraction after HSV-1 infection, a simple explanation is that USP44 interacts with MITA and is recruited to membranes following viral infection.

Several lines of evidence suggest that USP44 deconjugates K48-linked polyubiquitin from K236 of MITA after viral infection, which in turn inhibits proteasomal degradation of MITA. First, USP44 deubiquitinated MITA both *in vitro* and *in vivo*. Second, USP44 specifically removed K48- but not other linkage-mediated polyubiquitinaion of MITA. Third, USP44-deficiency increased HSV-1-induced K48-polyubiquitination and degradation of MITA, which was rescued by the proteasomal inhibitor MG132. Fourth, deubiquitination assays with MITA and its KR mutants showed that MITA(K236R) resisted USP44-mediated deubiquitination. Previous studies have reported that E3 ligases RNF5, TRIM30α and TRIM29 mediate K48-linked polyubiquitination of MITA at K150, K275, K288, K337 and K370 [33, 45–47], however, the E3 ligase responsible for MITA-ubiquitination at K236 remains to be identified.

Previously, it has been demonstrated that USP13 removes K27-linked polyubiquitin chains from MITA and impairs its recruitment of TBK1, contributing to inhibition of innate immune response to DNA viruses [36]. It has also been reported that USP20 deconjugates K33- and K48-linked polyubiquitin chains from MITA thus maintains its stability [48, 50]. Interestingly, USP20 has also been shown to deubiquitinate and stabilize ULK1 which is a negative regulator of MITA [51, 52]. Recently, it has been reported that CYLD removes K48-linked polyubiquitination from MITA at K150 [49]. However, CYLD-mediated deubiquitination lacks target specificity in the regulation of innate immune response as it has also been shown to deubiquitinate TRAF2, RIG-I and TBK1 [53–56]. Importantly, reconstitution experiments demonstrated that USP44-mediated regulation of MITA signaling is independent of USP20 and CYLD. In light of these and our studies, it is possible that MITA is distinctly regulated by different USPs in a spatial and temporal manner, so that it could be properly activated and inactivated at the onset and termination of innate immune response to DNA viruses.

Previous studies have shown that USP44 plays roles in tumorigenesis, cell cycle and DNA damage response [39, 40, 42, 43, 57]. It has also been shown that the DNA damage factors, meiotic recombination 11 homolog A (MRE11) and Ku70/80 complexes play important roles in initiation of dsDNA-induced type I IFN production [58, 59]. It would be interesting for the future studies to investigate whether USP44 is involved in the cross-talk between DNA damage and innate immune response.

## Materials and methods

### Reagents, antibodies, viruses and cells

Dual-specific luciferase assay kit (Promega); RNase inhibitor, lipofectamine 2000 (Thermo); GeneMute siRNA Transfection Reagent (SignaGen Laboratories); SYBR (Bio-Rad); polybrene (Millipore); 2’3’-cGAMP (InvivoGen); recombinant IFN-β (R&D Systems); HSV120, ISD (Sangon Biotech); and ELISA kits for murine IFN-β and IL-6 (BioLegend) and CXCL10 (Boster) were purchased from the indicated manufacturers.

Rabbit monoclonal antibodies against p-TBK1, p-IRF3, p-IKKβ, p-p65, MITA (Cell Signaling Technology); TBK1 (Abcam); β-actin (Abclonal); mouse monoclonal antibodies against Flag (Sigma); HA (OriGene); and ubiquitin (Santa Cruz Biotehcnology) were purchased for the indicated manufacturers.

HSV-1 (KOS strain) was purchased from China Center for Type Culture Collection. Human embryonic kidney (HEK293T) (Cat # CRL-11268) and human peripheral blood monocyte (THP-1) (Cat # TIB-202) cells were purchased from ATCC. Human foreskin fibroblasts (HFFs) were provided by Dr. Min-Hua Luo (Wuhan Institute of Virology).

### Constructs

Expression plasmids for USP20-His, CYLD-His, USP44-His, USP20-Flag, CYLD-Flag, USP44-Flag, USP44-Myc and its mutant were constructed by standard molecular biology techniques. Expression plasmids for HA-tagged cGAS, MITA, TBK1, IRF3, ubiquitin and ubiquitin mutants, and Flag-tagged MITA, MITA mutants, and the *IFNB* promoter, ISRE and NF-κB reporter were previously described [60–63].

### Transfection and reporter assays

HEK293T cells were transfected using a standard calcium phosphate precipitation protocol as previously described [64–66].

### Stable cell lines

The HEK293T cells were transfected with two packaging plasmids (pGAG-Pol and pVSV-G) together with empty vector, or the indicated plasmids respectively by calcium phosphate precipitation. Twenty-four hours later, medium was replaced. Fourty-eight hours later, the recombinant virus-containing medium was then filtered with 0.45 μm filter and added to THP-1 or iMLF cells in the presence of polybrene (8 μg/mL). Twenty-four hours post infection, cells were selected with puromycin (1 μg/mL) for 7 days before experiments.

### qPCR

Total RNA was isolated for qPCR analysis to measure mRNA levels of the indicated genes as previously described [63, 64, 67–69]. Data shown are the relative abundance of the indicated mRNA derived from human or mouse cells normalized to that of GAPDH. Primer sequences for qPCR assays are:

Human GAPDH: Forward-GACAAGCTTCCCGTTCTCAG;
Reverse-GAGTCAACGGATTTGGTGGT.
Human USP44: Forward-CGTATGTGACCAGTGTAA;
Reverse-GTGGCATATCATAAGTTGTT.
Human IFNB1: Forward-TTGTTGAGAACCTCCTGGCT;
Reverse-TGACTATGGTCCAGGCACAG.
Human IFIT1: Forward-TCATCAGGTCAAGGATAGTC;
Reverse-CCACACTGTATTTGGTGTCTACG.
Human IL6: Forward-GCCGCATCGCCGTCTCCTAC;
Reverse-CCTCAGCCCCCTCTGGGGTC.
Human CXCL10: Forward-GGTGAGAAGAGATGTCTGAATCC;
Reverse-GTCCATCCTTGGAAGCACTGCA.
Murine Gapdh: Forward-ACGGCCGCATCTTCTTGTGCA;
Reverse-ACGGCCAAATCCGTTCACACC.
Murine Usp44: Forward-CCACTTCCCAAAGGAGACTTAT;
Reverse-CACTGGTATCTCTCTGGAAACTC.
Murine Ifnb1: Forward-TCCTGCTGTGCTTCTCCACCACA;
Reverse-AAGTCCGCCCTGTAGGTGAGGTT.
Murine Ifit1: Forward-ACAGCAACCATGGGAGAGAATGCTG;
Reverse-ACGTAGGCCAGGAGGTTGTGCAT.
Murine Il6: Forward-TCTGCAAGAGACTTCCATCCAGTTGC;
Reverse-AGCCTCCGACTTGTGAAGTGGT.
Murine Cxcl10: Forward-ATCATCCCTGCGAGCCTATCCT;
Reverse-GACCTTTTTTGGCTAAACGCTTTC.
HSV-1 ICP22: Forward-TGTTTGGAGACCAGACGGTA;
Reverse-CATCGGAGATTTCATCATCG.
HSV-1 ICP27: Forward-GGCCTGATCGAAATCCTAGA;
Reverse-GTCAACTCGCAGACACGACT.

### Co-immunoprecipitation and immunoblot analysis

HEK293T cells were lysed in 1mL NP-40 lysis buffer (20 mM Tris-HCl pH 7.4, 150 mM NaCl, 1 mM EDTA, 1% NP-40, 10 μg/ml leupeptin, 1 mM phenylmethylsulfonyl fluoride). For each immunoprecipitation, 0.4 ml aliquot of the lysate was incubated with control IgG or the indicated antibody (0.5 μg) and 15 μL of Protein G Sepharose (GE Healthcare) at 4°C for 3 h or overnight. The Protein G sepharose beads were washed three times with 1 mL of lysis buffer containing 0.5 M NaCl. Immunoblot analysis was performed by standard procedures.

### RNA interference

Double-stranded oligonucleotides corresponding to the targeting sequences were cloned into the pSuper-Retro-RNAi plasmid (Oligoengine) as previously described [70–72]. The following sequences were targeted for Human USP44-RNAi: #1 5’- GTAACAGGATTGAGAAATT-3’, #2 5’- GGAATTTCTTTGTGAACTT-3’; Human USP44-siRNA: 5’- GAATTGGAGTATCAAGTTA-3’. The siRNA was delivered into HFF cells by GeneMute siRNA Transfection Reagent (SignaGen Laboratories) according to the protocol provided by the manufacturer.

### Preparations of BMDMs and MLFs

Bone marrow cells were isolated from tibia and femur. For preparation of bone marrow-derived macrophages (BMDMs), bone marrow cells were cultured in 10% M-CSF-containing conditional medium for 5 days. For murine lungs fibroblasts (MLFs), lungs were minced and digested in calcium and magnesium free HBSS containing 10 μg/ml type II collagenase and 20 μg/ml DNase I for 1 h at 37°C with shaking. Cell suspension was centrifuged at 500 *g* for 5 min. The cells were then plated in culture medium (1:1 [v/v] DMEM/Ham’s F-12 containing 10% fetal bovine serum (FBS), 50 U/ml penicillin, 50 μg/ml streptomycin, 15 mM HEPES, 2 mM _L_-glutamine). For generation of immortalized MLFs, MLFs were infected with SV40 in the presence of polybrene (8 μg/mL), 24 h later cells were cultured with fresh medium and immortalized cells were selected.

### *Usp44*^*-/-*^ mice

The genotype of the *Usp44*^*-/-*^ mice was confirmed by sequencing PCR products amplified from the genomic DNA isolated from mouse tails using the following primers: #1: 5’- CTCCAATTCCGATCATATTCAATAAC-3’, #2: 5’-CCTCATCCCCACTTCCCAAAGGAG-3’ and #3: 5’-CTCTCTCCCCCATAAAGCCACTGC-3’.

### HSV-1 infection of mice

Eight-week-old *Usp44*^*+/+*^ and *Usp44*^*-/-*^ mice were intra-peritoneally (i.p.) or intra-nasally (i.n.) infected with HSV-1. The serum was collected at 6 h post infection for measurement of IFN-β, IL-6 and CXCL10 by ELISA. The viability of the infected mice was monitored for 9 days.

### Plaque assay

The brain from HSV-1 infected mice were collected 5 days post infection. The brains were weighed and homogenized with PBS, followed by centrifugation at 1620 *g* for 30 min, and the supernatants were collected for plaque assays as previously described [60, 62, 67].

### Subcellular fractionation

HEK293T cells were transfected with USP44-Flag for 24 h, nuclear and cytoplasmic fractions were extracted using NE-PER Nuclear and Cytoplasmic Extraction Reagents Kit (Thermo 78835).

For isolation of membraneous and cytosolic fractions, cells were washed with PBS and lysed by bouncing for 50 times in a homogenization buffer (10 mM Tris-HCl [PH 7.4], 10 mM potassium chloride, 2 mM magnesium chloride and 250 mM saccharose). The homogenate was centrifuged at 500 *g* for 10 min and the supernatant was centrifuged at 5000 *g* for 10 min. The supernatant from this step was further centrifuged at 20000 *g* for 30 min. The pellet was membraneous fraction and the supernatant was cytosol.

### Deubiquitination assays

Cells were lysed with the lysis buffer (100 μl) and the supernatants were denatured at 95°C for 5 min in the presence of 1% SDS. The denatured lysates were diluted with NP-40 lysis buffer until the concentration of SDS was reduced < 0.1% followed by immunoprecipitation with the indicated antibodies.

### *In vitro* deubiquitination assays

Denature-IP was performed to obtain ubiquitin-modified MITA from HEK293T cells co-transfected with Flag-tagged MITA and HA-Ubiquitin. The immunoprecipitates were eluted by Flag peptide (0.5 mg/ml, 60 μl). USP44 or USP44(C282A) protein was obtained utilizing an in vitro transcription and translation kit (Promega). The ubiquitin-modified Flag-MITA was incubated with USP44 or USP44(C282A) at 37°C for 2 h followed with overnight incubation at 16°C in the presence of ATP (1 μM). The mixtures were analyzed by immunoblots with the indicated antibodies.

### Ethics statement

All mice were housed in the specific pathogen-free facility and viral infection experiments were carried out in an ABSL-2 facility at Wuhan Institute of Virology. The experimental protocol was adhered to the International Guiding Principles for Biomedical Involving Animals. The protocol for animal experiments were approved by the Institutional Animal Care and Use Committee of Wuhan Institute of Virology (approval number WIVA31201601).

### Statistical analysis

Student’s t test was used for statistical analysis with Microsoft Excel and GraphPad Prism Software, P<0.05 was considered significant. For the mouse survival study, Kaplan-Meier survival curves were generated and analyzed by Log-Rank test.

## Supporting information

S1 Fig Identification of USP44 as a positive regulator of DNA virus-triggered signaling. HEK293 cells (1 x 10^5^) were co-transfected with the *IFNB* promoter (0.05 μg) and empty vector or independent human DUB cDNA expression plasmids (0.05 μg) for 24 hours. Cells were then left uninfected or infected with HSV-1 (MOI = 1) for 12 hours before luciferase assays were performed. Graphs show mean ± S.D. n = 3. *P < 0.05, **P < 0.01 (Student’s *t*-test).
(TIF)

S2 Fig Knockdown of USP44 inhibits cytosolic DNA-induced signaling. THP-1 cells (4 x 10^5^) stably expressing USP44-RNAi were transfected with ISD (2 μg/ml) for 12 hours before qPCR analysis. Graphs show mean ± S.D. n = 3. *P < 0.05, **P < 0.01 (Student’s *t*-test).
(TIF)

S3 Fig USP44 is required for cytosolic DNA-triggered induction of antiviral genes. **(A)** Genotyping of *Usp44* knockout mice. **(B)** BMDMs (4 x 10^5^, left) or MLFs (4 x 10^5^, right) were left uninfected or infected with HSV-1 (MOI = 1) for the indicated times, followed by qPCR analysis. **(C)** BMDMs (4 x 10^5^) were left uninfected or infected with VACV for the indicated times before qPCR analysis. **(D)** BMDMs (4 x 10^5^) were transfected with ISD (2 μg/ml) for the indicated times before qPCR analysis. **(E)** MLFs (4 x 10^5^) were infected with HSV-1 (top) (MOI = 1) or transfected with HSV120 (middle) or ISD (bottom) (2 μg/ml) for the indicated times before qPCR analysis were performed. **(F)** MLFs (4 x 10^5^) were left uninfected or infected with SeV (MOI = 1) for 6 h before qPCR analysis. **(G)** MLFs (4 x 10^5^) were infected with HSV-1 (MOI = 1) for the indicated times, followed by immunoblot with the indicated antibodies. Graphs show mean ± S.D. n = 3. *P < 0.05, **P < 0.01 (Student’s *t*-test).
(TIF)

S4 Fig USP44 was detected in both the nuclear and cytoplasmic fractions. HEK293T cells (2 x 10^6^) were transfected with USP44-Flag plasmids for 24 h, followed by nuclear and cytoplasmic extraction.
(TIF)

S5 Fig USP44-mediated regulation of MITA is independent of USP20 or CYLD. **(A)** HEK293T cells (1 x 10^5^) were co-transfected with empty vector or cGAS and MITA, IFN-β (0.05 μg), ISRE (0.05 μg) or NF-κB (0.01 μg) reporter plasmids, and the indicated plasmids for 24 hours before luciferase assays were performed. **(B)** HEK293T cells (1 x 10^6^) were transfected with the indicated plasmids for 24 hours before deubiquitination assays were performed.**(C)** iMLF stable cell lines (4 x 10^5^) were left uninfected or infected with HSV-1 (MOI = 1) for the indicated times before qPCR analysis. Graphs show mean ± S.D. n = 3. *P < 0.05, **P < 0.01 (Student’s *t*-test).
(TIF)
