# Supplementary figures and images for: USP44 positively regulates innate immune response to DNA viruses through deubiquitinating MITA
Source: PLoS Pathog. 2020 Jan 22;16(1):e1008178. doi: 10.1371/journal.ppat.1008178 (PMC6975528; doi:10.1371/journal.ppat.1008178)

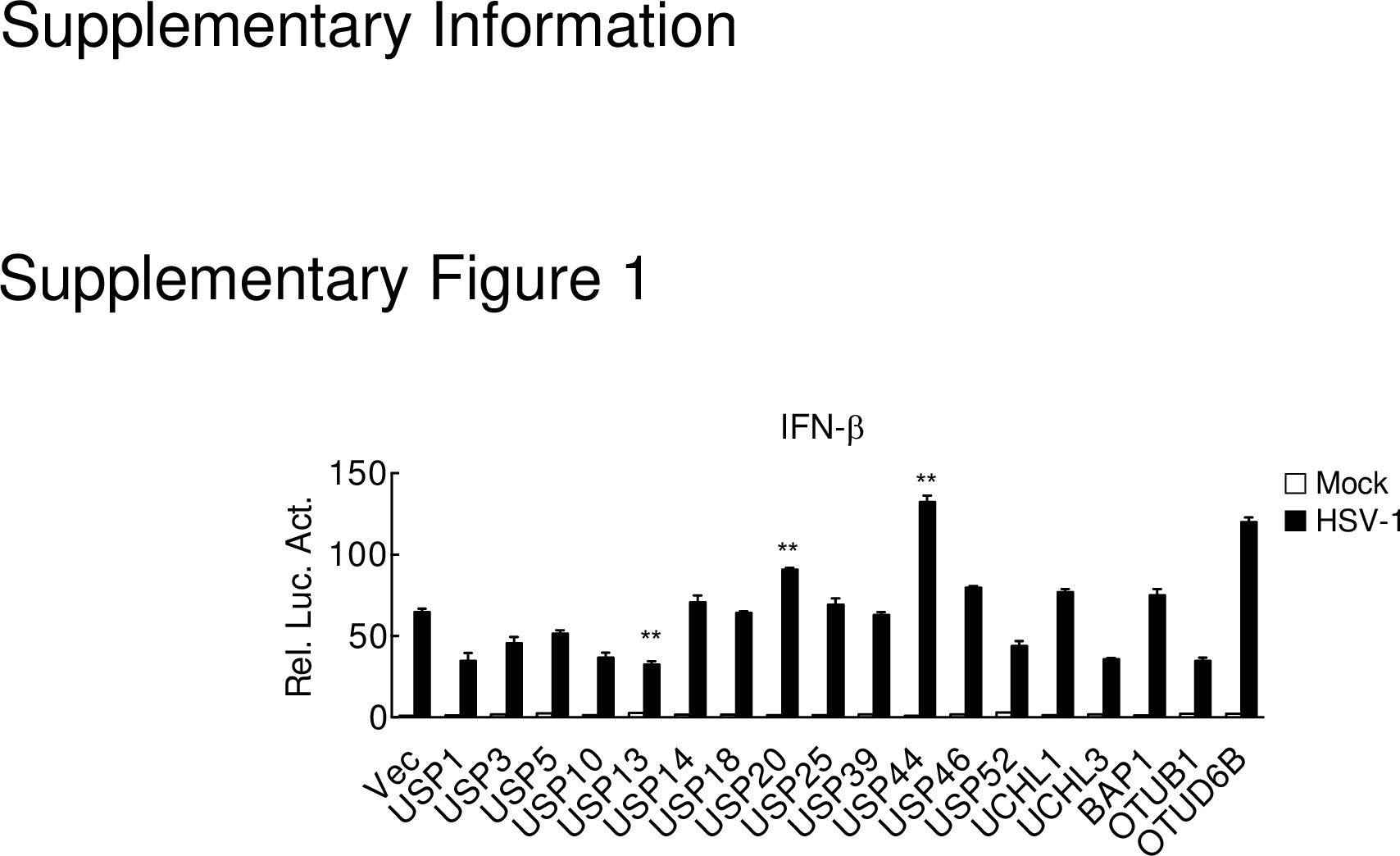

Supplement: S1 Fig — HEK293 cells (1 x 10^5^) were co-transfected with the IFNB promoter (0.05 μg) and empty vector or independent human DUB cDNA expression plasmids (0.05 μg) for 24 hours. Cells were then left uninfected or infected with HSV-1 (MOI = 1) for 12 hours before luciferase assays were performed. Graphs show mean ± S.D. n = 3. *P < 0.05, **P < 0.01 (Student’s t-test). (TIF) [file ppat.1008178.s001.tif]

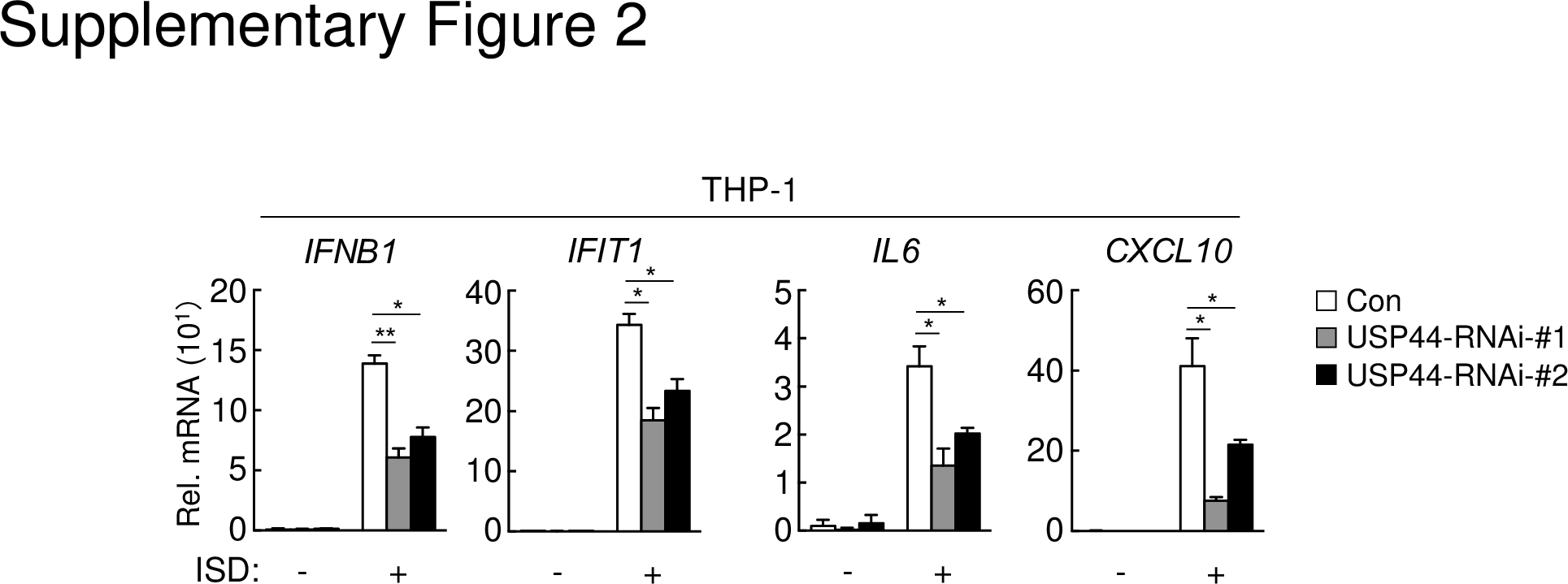

Supplement: S2 Fig — THP-1 cells (4 x 10^5^) stably expressing USP44-RNAi were transfected with ISD (2 μg/ml) for 12 hours before qPCR analysis. Graphs show mean ± S.D. n = 3. *P < 0.05, **P < 0.01 (Student’s t-test). (TIF) [file ppat.1008178.s002.tif]

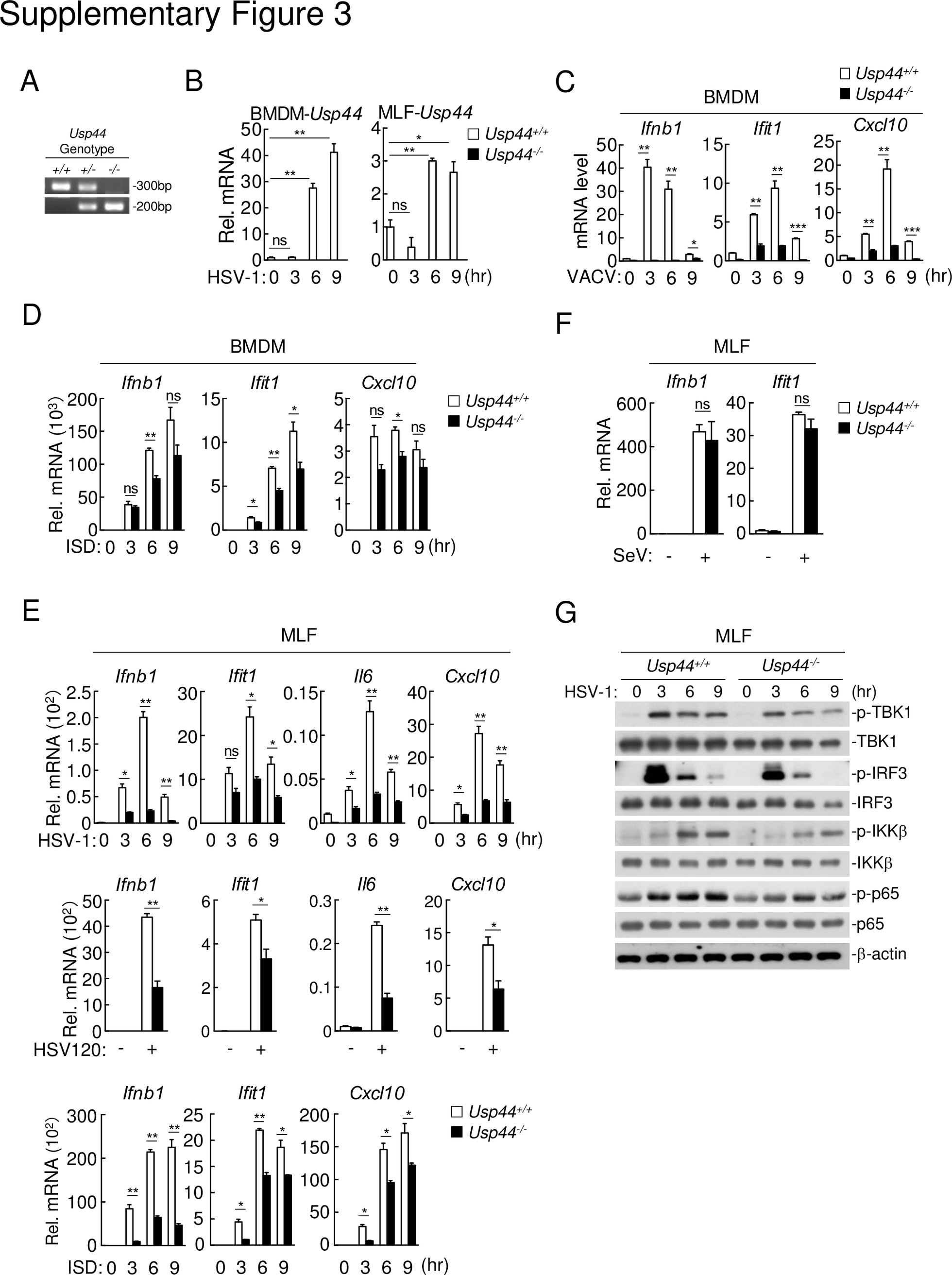

Supplement: S3 Fig — (A) Genotyping of Usp44 knockout mice. (B) BMDMs (4 x 10^5^, left) or MLFs (4 x 10^5^, right) were left uninfected or infected with HSV-1 (MOI = 1) for the indicated times, followed by qPCR analysis. (C) BMDMs (4 x 10^5^) were left uninfected or infected with VACV for the indicated times before qPCR analysis. (D) BMDMs (4 x 10^5^) were transfected with ISD (2 μg/ml) for the indicated times before qPCR analysis. (E) MLFs (4 x 10^5^) were infected with HSV-1 (top) (MOI = 1) or transfected with HSV120 (middle) or ISD (bottom) (2 μg/ml) for the indicated times before qPCR analysis were performed. (F) MLFs (4 x 10^5^) were left uninfected or infected with SeV (MOI = 1) for 6 h before qPCR analysis. (G) MLFs (4 x 10^5^) were infected with HSV-1 (MOI = 1) for the indicated times, followed by immunoblot with the indicated antibodies. Graphs show mean ± S.D. n = 3. *P < 0.05, **P < 0.01 (Student’s t-test). (TIF) [file ppat.1008178.s003.tif]

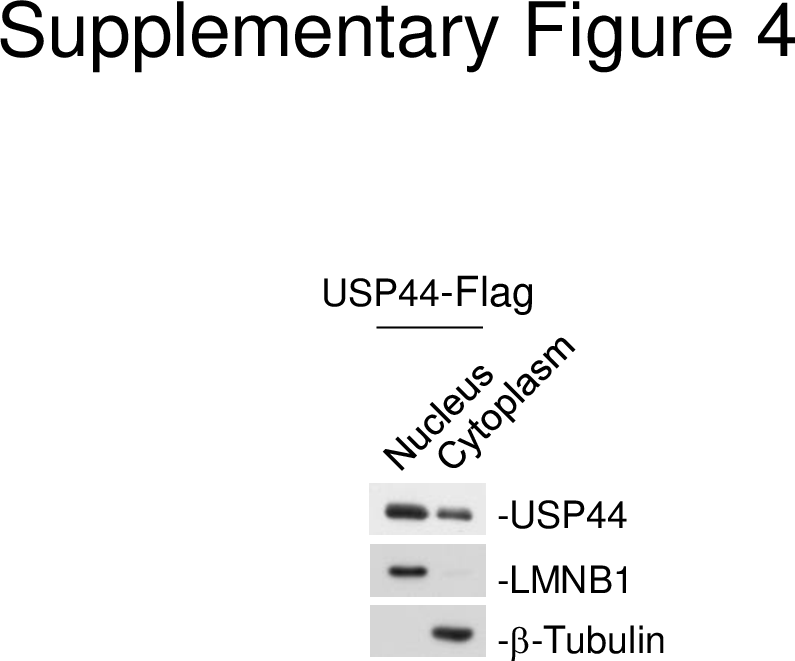

Supplement: S4 Fig — HEK293T cells (2 x 10^6^) were transfected with USP44-Flag plasmids for 24 h, followed by nuclear and cytoplasmic extraction. (TIF) [file ppat.1008178.s004.tif]

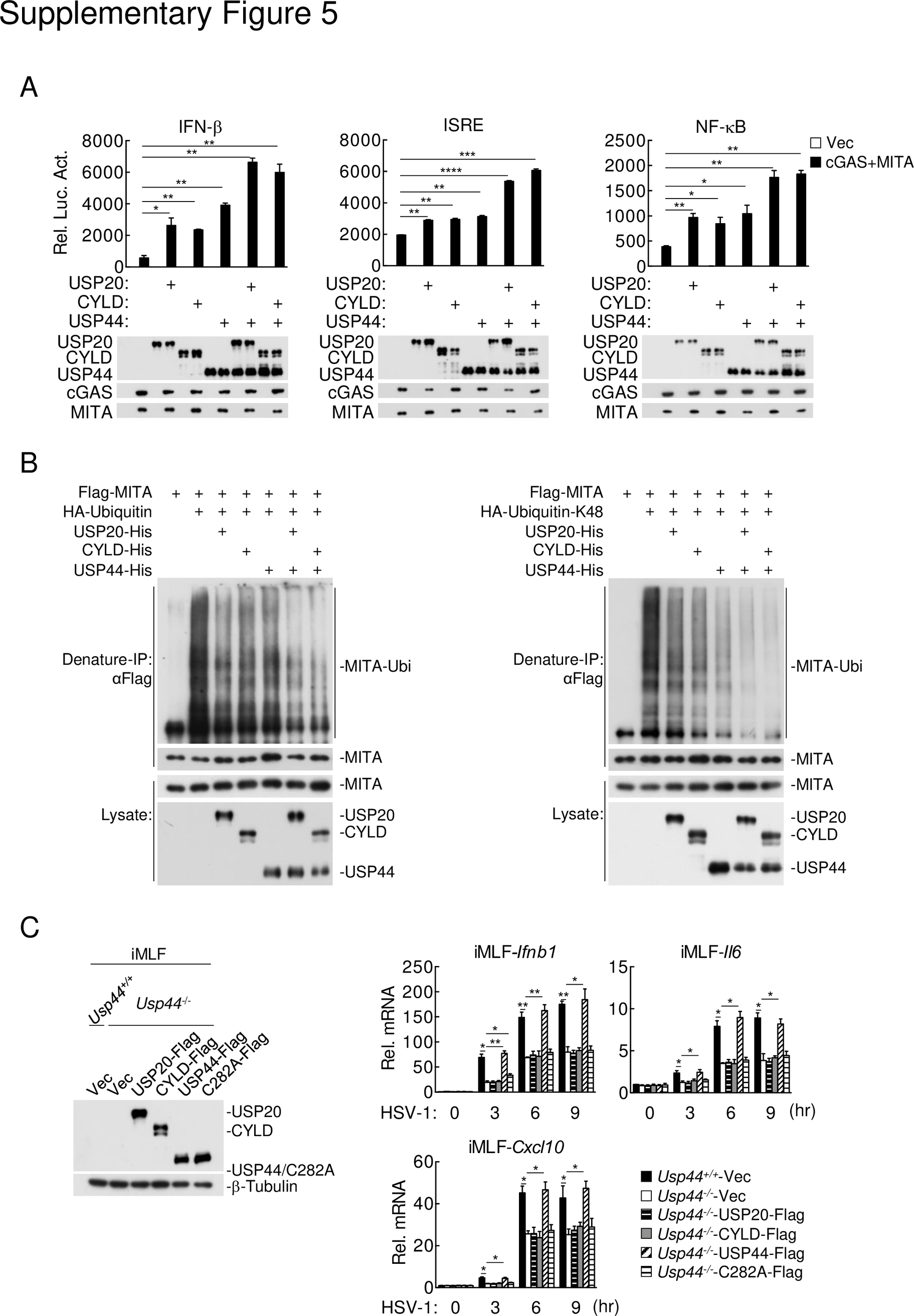

Supplement: S5 Fig — (A) HEK293T cells (1 x 10^5^) were co-transfected with empty vector or cGAS and MITA, IFN-β (0.05 μg), ISRE (0.05 μg) or NF-κB (0.01 μg) reporter plasmids, and the indicated plasmids for 24 hours before luciferase assays were performed. (B) HEK293T cells (1 x 10^6^) were transfected with the indicated plasmids for 24 hours before deubiquitination assays were performed.(C) iMLF stable cell lines (4 x 10^5^) were left uninfected or infected with HSV-1 (MOI = 1) for the indicated times before qPCR analysis. Graphs show mean ± S.D. n = 3. *P < 0.05, **P < 0.01 (Student’s t-test). (TIF) [file ppat.1008178.s005.tif]
